# Endoscopic Sinus Surgery in Frontal Sinus Inverted Papilloma: A Systematic Review

**DOI:** 10.3390/jpm15050183

**Published:** 2025-05-02

**Authors:** Maxime Fieux, Valentin Favier, Andre Sousa Machado, Mikail Nourredine, Caroline Giroudon, Florent Carsuzaa, Paresh P. Naik, the yo-IFOS Group

**Affiliations:** 1 Hospices Civils de Lyon, Centre Hospitalier Lyon Sud, Service d’ORL, d’Otoneurochirurgie et de Chirurgie Cervico-Faciale, 69495 Pierre Bénite, France; 2 Université de Lyon, Université Lyon 1, 69003 Lyon, France; 3 Département d’ORL, Chirurgie Cervico Faciale et Maxillo-Faciale, Hôpital Gui de Chauliac, CHU de Montpellier, 34295 Montpellier, France; valentin_favier@hotmail.com; 4 Research-Team ICAR, Laboratory of Computer Science, Robotics and Microelectronics of Montpellier (LIRMM), Université de Montpellier, French National Centre for Scientific Research (CNRS), 34095 Montpellier, France; 5 Department of Otolaryngology, Unidade Local de Saúde de Trás-os-Montes e Alto (ULSTMAD), CP 5000-508 Vila Real, Portugal; sousamachado.andre@gmail.com; 6 Faculty of Health Sciences, University of Beira Interior, CP 6200-506 Covilhã, Portugal; 7 Service de Biostatistiques de Lyon, Pôle Santé Publique, Hospices Civil de Lyon, 69002 Lyon, France; mikail.nourredine@chu-lyon.fr; 8 Service de la Documentation Centrale, Hospices Civils de Lyon, 69424 Lyon, France; caroline.giroudon@chu-lyon.fr; 9 Service ORL, Chirurgie Cervico-Maxillo-Faciale et Audiophonologie, Centre Hospitalier Universitaire de Poitiers, 86000 Poitiers, France; florent.carsuzaa@gmail.com; 10 Laboratoire Inflammation Tissus Epithéliaux et Cytokines (LITEC), UR15560, Université de Poitiers, 86000 Poitiers, France; 11 Jaslok Hospital & Research Centre, Pedder Road, Mumbai, Maharashtra 400026, India; pareshnaikp@gmail.com

**Keywords:** sinus surgery, endoscopic sinus surgery, inverted papilloma, frontal sinus, recurrence

## Abstract

**Background**: Frontal sinus inverted papilloma (IP) is a particularly rare form of IP and its management is challenging, with a high rate of recurrence. **Objectives**: Our aim was to evaluate the recurrence rate of frontal sinus IP after surgery and compare this rate according to the surgical modality (purely endoscopic sinus surgery vs. a combined/open approach). **Design**: A systematic review without meta-analysis conducted by a working group of the Young Otolaryngologists of the International Federation of Otorhinolaryngological Societies (yo-IFOS). **Data Sources and Methods**: A systematic analysis of the literature was performed and reported following the criteria laid down in the SWiM guidelines. The review was registered on Prospero, a dedicated software was used for screening (Covidence), and R (v.4.2.2) was used for statistical analysis. Eligible articles were studies reporting at least five cases of frontal sinus IP surgically treated. **Results**: A total of 2925 studies were identified based on the MeSH equation, and 39 studies were included (n = 642 patients). Among the studies included, the recurrence rate was 18.4% (118/642) with a mean time to recurrence of 25.6 (±11.7) months. The difference between surgical modalities was not statistically significant in terms of recurrence rate (14.7% vs. 16.5%; *p* = 0.675). **Conclusions**: The recurrence rate of frontal sinus IP is not different between surgical modalities. However, it does not reduce the need for a tailored therapeutic strategy, as other factors also need to be considered (time to recurrence, complications, quality of life) when choosing the most appropriate approach.

## 1. Introduction

Sinonasal inverted papilloma (IP) is a rare benign neoplasm accounting for 0.5–4% of nasal sinus neoplasms [1,2]. There is a male predominance, a male-to-female ratio of 3.4:1, and a mean age of 55 years [3], and an incidence of 0.01 to 0.33/100,000 person–years [4]. Although smoking, viral infections, diabetes, allergies, and chronic inflammation are possible causes, human papillomavirus (HPV) is considered a significant cofactor in the pathogenesis—especially types 6, 11, 16, and 18 [5,6]. Recent reports have also suggested that occupational exposure is a risk factor for sinonasal IP [7]. Sinonasal IP has a recurrence rate of 15–30%, with locally aggressive features that lead to the destruction of adjacent bony structures and the compression of neurovascular adjacent structures. Recurrence is defined here as any positive imaging or endoscopic abnormalities where biopsies were performed and recurrence histologically proven. It is also known that IP is associated with malignant transformation, with a rate of transformation into squamous cell carcinoma (SCC) between 7 and 9% [3,8]. As a result, an extended surgical excision is essential, requiring the removal and drilling of the tumor’s bony attachment point [9]. Sinonasal IP treatment has evolved from an open surgery to a purely endoscopic sinus surgery. The most frequent site of attachment is the lateral nasal wall, followed by the maxillary and ethmoid sinuses and walls. Less commonly, IP can arise in the frontal sinus, nasal septum, or sphenoid sinus [10,11]. The size and origin of the tumor can be preoperatively determined using imaging with an accuracy of 74–90%. They are both of the utmost importance for surgical planning and success [10].

Frontal sinus IP is a particularly rare form of this condition, representing only 2.5–5% of all IP cases [12,13]. Frontal sinus IP is suspected to carry a higher likelihood of recurrence compared to IP arising in other areas of the sinonasal cavity [12,14]. However, there is currently no robust data in the literature to support this point. Indeed, most authors use the Krouse classification [15], which gathers the frontal, sphenoid, and non-medial maxillary wall locations in stage III. When feasible, a purely endoscopic sinus surgery is the preferred treatment method for frontal sinus IP. This is due to its favorable outcomes and lower morbidity compared to more invasive open surgery [16]. Some challenging anatomical factors, such as limited access to the tumor’s pedicle, may dictate a combined surgical approach (endoscopic and open approach). This kind of combined approach enables its total removal and regional control [11].

The aim of this systematic review, conducted by a working group of the Young Otolaryngologists of the International Federation of Otorhinolaryngological Societies (YO-IFOS), was to evaluate the recurrence rate of frontal sinus IP after surgery and compare this rate according to the surgical modality (purely endoscopic sinus surgery or combined/open approach).

## 2. Materials and Methods

### 2.1. Synthesis Without Meta-Analysis Criteria

The systematic analysis of the literature was performed and reported following the criteria laid down in the Synthesis Without Meta-Analysis guidelines (SWiM) [17] and the PRISMA checklist (attached) to ensure that the research strategy and statistics would be reproductible.

### 2.2. Protocol and Registration

The study was registered in the Prospero database in April 2024 (n° CRD42023487678) as a systematic review of health research studies including human subjects.

### 2.3. Inclusion Criteria

Eligible articles were articles reporting original clinical or preclinical research on modalities, effectiveness, and the safety of surgical treatment of frontal sinus IP, published in the medical literature at any time between 1960 and 2024. Although the inclusion period began in 1960, we performed a sensitivity analysis focused on studies published after 2000 to assess consistency with modern surgical techniques. Only articles reporting the main outcome (i.e., the exact number of recurrences from IP emerging from the frontal sinus) were included. The human studies included randomized controlled trials (RCTs), non-randomized controlled trials, and observational studies. Systematic analyses, with or without meta-analyses, narrative analyses, and case reports of a single patient were excluded. Reviews, although not included, were examined individually to analyze their reference lists for articles liable to meet the inclusion criteria.

### 2.4. Sources

Studies were identified in the Pubmed/Medline (US National Library of Medicine), Web of Science (Clarivate), CENTRAL, SciELO and Embase (Elsevier) databases. They were queried on 04/24/2024. Further references were sought in the articles’ reference lists. Automatic alerts were set up for each database to monitor the publication of studies likely to enrich the work during the writing and analysis phase. The literature watch was continued up to 15 March 2025 (submission).

### 2.5. Literature Search Strategy

The search formula was drawn up using Medical Subject Headings (MeSH) and free-text abbreviations and terms relating to allergic and non-allergic rhinitis. Keywords included but were not limited to “Papilloma, Inverted” AND “Frontal Sinus” OR “Paranasal Sinus Neoplasms” OR “Paranasal Sinuses”. We did not use a time limit in our research. Only studies published in the following languages were selected: English, French, Spanish, Italian, German, and Portuguese. The search formulae were defined by an information specialist of the Lyon university hospitals (C.G.); the MeSH and free-text equations are detailed in the Supplementary Material S1.

### 2.6. Article Selection

**Figure 1:**
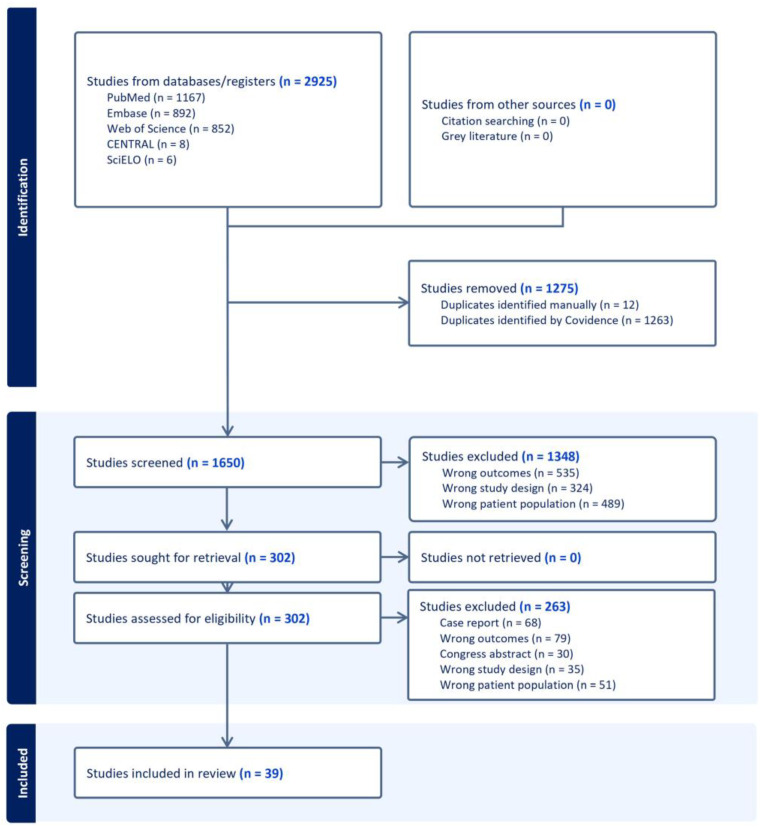
SWiM flowchart. Footnotes: The flowchart was automatically generated using the software Covidence, which enabled double-blind screening for both phases (title and abstract; full text).

Figure 1 shows the SWiM flowchart for article selection drawn up using Covidence, which was used to carry out the double-blind screening work. Retrieved citations and abstracts were compiled in a single reference list. The software eliminated duplicates. Selection proceeded in two phases: the selection of titles and abstracts (MF, PN), then selection on full text (VF, ASM). Relevant articles were examined in greater depth, and those not meeting the inclusion criteria were rejected. In case of disagreement, a third opinion was sought from another investigator (FC). If the data in the abstract were insufficient for a decision, the full text of the article was obtained and read. When full text was not available (presentations or lectures), an inter-library loan was requested and, if the full text was still not available, the article was excluded. The full-text articles included were examined to extract title, author, date, journal, number of references, type of study, surgical modality, sample size, gender, age, follow-up duration, type of intervention, Krouse classification, recurrence rate, time to recurrence, complications, and risk of bias; these data were entered in a standardized Microsoft Excel^®^ spreadsheet for systematic reviews.

### 2.7. Data Collection and Selection

Data were extracted from texts, tables, or graphs. International units were used, with conversion as necessary. Continuous data were used if possible, and dichotomous or categoric variables if not.

### 2.8. Risk of Bias Assessment Methods

The bias analysis was carried out by two investigators (MF, VF) using the Methodological Index for Non-Randomized Studies (MINORS) tool, which is a validated instrument designed for assessing the quality of non-randomized surgical studies [18]. The MINORS tool consists of 12 items related to the analysis of methodological points of comparative (ideal score = 24) and non-comparative studies (ideal score = 16).

### 2.9. Main Results

The main outcome was the proportion of recurrence after surgery in patients with frontal sinus IP. The secondary outcome was the comparison of recurrence rate according to the surgical modality, i.e., purely endoscopic sinus surgery or other surgical approach. The latter was defined herein as either open surgery alone or open and endoscopic surgery combined. First, the demographics, surgical modalities, type of intervention (primary or revision surgery), quality of life scores, safety (minor and major adverse events), and tumor characteristics (Krouse classification) of the included studies were described. A minimum of 5 studies was considered sufficient to perform such a descriptive analysis. Then, the main and secondary outcomes were reported. Finally, the risk of bias was assessed. Data analysis was performed using R software (version 4.3.3).

## 3. Results

### 3.1. Study Selection

A total of 2925 studies were identified based on the MeSH equation; 302 were assessed for full-text screening after duplicates (n = 1275) and non-eligible studies based on the title and abstract were excluded (n = 1348, Figure 1). Finally, 39 studies (n = 642 patients), all observational, were included in the analysis—34 of these met the initial inclusion criteria and 5 were added based on the literature update pursued until submission. The majority of included studies (97.4%) were published after 2000 (only 1 was published in 1995).

### 3.2. Description of Included Studies

Among the 39 studies included [12,14,16,19,20,21,22,23,24,25,26,27,28,29,30,31,32,33,34,35,36,37,38,39,40,41,42,43,44,45,46,47,48,49,50,51,52,53,54], 16 (±14) patients per study were included in average, at a mean age of 55.5 (±3.5) years old, and for a mean follow-up of 39.8 (±17.9) months. There was a male-to-female ratio favoring males, with 70.4% male patients (152/216; data available for 6 studies) [16,22,41,47,49,51]. Regarding surgical modality, data were available for 64.0% of studies (25/39; n = 485 patients); a total of 71.3% (346/485) of patients underwent purely endoscopic sinus surgery and the other 28.7% (139/485) underwent other approaches (Table 1). Type of intervention (primary or revision surgery), quality of life, safety, and Krouse classification could not be included in the analysis because of a lack of data regarding the specific frontal sinus location (less than 5 studies had available data).

### 3.3. Recurrence Rate After Surgery in Frontal Sinus IP

Among the 39 studies included, the recurrence rate was 18.4% (118/642) with a mean time to recurrence of 25.6 (±11.7) months. Among patients who underwent purely endoscopic sinus surgery, the recurrence rate was 14.7% (51/346) compared to 16.5% (23/139) in patients who underwent other approaches (*p* = 0.675). Regarding time to recurrence, which was available in five studies [19,20,30,39,49], it was 28.0 (±11.3) months in patients who underwent a purely endoscopic sinus surgery and 73.2 (±40.8) months in patients who underwent other approaches with no significant difference (*p* = 0.095; Table 1).

### 3.4. Risk of Bias Assessment

Among the 39 studies included, 25 had a control group and were considered as comparative studies. The mean MINORS score for all studies included was 13.1 (±2.8). Among the comparative studies (n = 25), the mean MINORS score was 15.1/24 (±0.99), and among the non-comparative ones (n = 14), the mean MINORS score was 9.6/16 (±0.93; Table 2). 

## 4. Discussion

This systematic review included 39 studies corresponding to 642 patients, representing one of the largest systematic reviews to date with a focus on frontal sinus IP. The overall recurrence rate was 18.4%, and although it was not statistically different, the recurrence rate appeared to be lower among patients who underwent a purely endoscopic sinus surgery (14.7%) compared to those with other approaches (16.5%).

The demographics of the population suffering from frontal sinus IP described herein was similar to that of patients with IP of other locations, with a mean age of 55 years at diagnosis, as described in other locations (maxillary, ethmoid, or others), and a difference in male-to-female ratio [3,4,12]. However, the latter might be subject to sampling bias, as data were missing for 33 studies and should thus be considered with caution.

In the present study, the mean follow-up was 40 months with a recurrence rate of 14.7% for the purely endoscopic sinus surgery group, which is comparable to the described recurrence rate of 15% after endoscopic resection for IP of other locations [55]. The results herein are concordant with the literature. In a recent review of the literature on the long-term follow-up of IP, Kuan et al. found a recurrence rate of 16.1% (±9.1%) with a mean time to recurrence ranging from 8 to 45.5 months [11]. One of the hypotheses supporting this result is the role of endoscope magnification, which helps to clean the surgical field as easily as during an open approach in carefully selected patients [56].

In the present study, the mean recurrence rate of frontal sinus IP was 18.7% (121/647) with no significant difference found between purely endoscopic and combined approaches. Similarly, Gaudioso et al. recently reviewed the surgical outcomes of frontal IP [16]. They included both frontal sinus and frontal recess locations of the pedicle and found a similar 18.9% rate of recurrence. However, they found a higher recurrence rate when combined approaches were performed without osteoplastic flaps (not studied herein). This difference could be explained by how the authors grouped frontal sinus IP. Authors believe that given the widespread use of endoscopic sinus surgery even for extended approaches, purely intra-frontal and frontal recess IP should be distinguished, as the second one is not so difficult to access. Further data are needed as, to date, the difference is not clear within the available literature.

In most studies, the mode of frontal sinus involvement (i.e., bulging tumor or tumor pedicle) was not reported. Kim et al. found 17 cases of frontal sinus IP in a Korean multicenter study involving 939 patients, and among them, the frontal sinus was the origin site in 3% of cases, but the frontal sinus was involved in 16.6% of cases [14]. A more precise description of the IP implantation site should be performed to allow for a comparison between open/combined and purely endoscopic approaches for future studies. This description could be based on already existing classifications, such as the one on malignant tumors proposed by Bastier et al. [57]. The latter enables a precise description of the pedicle location. This detail is the most important factor in preventing the recurrence, as described by Adriaensen et al. [19]. Also, the completeness of resection in the primary endoscopic surgery is of course essential and should remain the main goal for every patient. The authors believe that the Krouse classification [15] is not up to date anymore, which is necessary to ensure a precise description of the origin site of the tumor in frontal sinus IP, and it could be one of the future aims of the European Position Paper on Sinus (EPOS).

Furthermore, several methodological limitations affect the strength of the evidence. There was no double-blind randomized controlled trials, and among the included studies, the number of studies with details regarding the surgical modalities were low. Due to inconsistent reporting of tumor staging, particularly the Krouse classification, we were unable to assess the potential impact of tumor stage on recurrence. This constitutes a major limitation in interpreting surgical outcomes. Overall, most of the studies analyzed had a high risk of bias. This risk is attributed to study designs and precisions on the surgical modalities and tumor location, which directly limits the strength and generalizability of our conclusions.

The authors wish to highlight that they conducted a comprehensive and rigorous search of literature on frontal sinus IP, which ultimately yielded 39 studies. However, the heterogeneity among these studies was high, both in methodology and in reporting, which inherently limits the strength of the conclusions one could draw. This is a recognized challenge in systematic reviews on rare and complex pathologies such as frontal sinus IP. The authors intentionally focused their analysis on endpoints that were consistently and explicitly reported across studies—namely, the surgical approach and recurrence rate. While many additional factors can influence recurrence, such as tumor extent, surgeon experience, and follow-up duration, the available data did not allow for a detailed subgroup analysis of these variables. Nonetheless, recurrence remains a clinically relevant endpoint that strongly influences surgical decision making, and we believe that synthesizing the currently available data provides value to the field. This clarification underscores our cautious interpretation of the findings and the incremental contribution of this systematic review to the ongoing discussion on optimal management strategies for frontal sinus IP. Different factors should be mentioned when performing a study on frontal sinus IP. Among them, the preoperative suspected location of the IP pedicle [58], the preoperative risk of malignant transformation [59], the reasons to choose an either purely endoscopic or combined approach (imaging data, tumor characteristics, and the experience of the surgeon), and the final location of the pedicle during the surgery are particularly important factors. Although, pre-operative informed consent from the patient is mandatory and it should be mentioned that even in case of an endoscopic approach, a possible conversion into an open approach might be needed [60].

## 5. Conclusions

Even if no robust conclusion could be drawn, it seems that the recurrence rate of frontal sinus IP is not different between surgical modalities. However, it does not reduce the need for a tailored therapeutic strategy, as other factors also need to be considered (time to recurrence, complications, quality of life) when choosing the most appropriate approach.

## Figures and Tables

**Table 1:** Comparison between surgical modalities.

Characteristics (n = 25 Studies)	Other Approaches	Purely Endoscopic	*p*-Value
Mean number of patients per study (±SD)	5.6 (7.0)	13.8 (15.2)	0.003
Age in years (±SD)	59.0 (5.6)	52.6 (2.8)	0.095
Follow-up in months (±SD)	40.2 (0.4)	30.7 (11.7)	0.381
Rate of recurrence	16.5%	14.7%	0.675
Time to recurrence; n = 5 studies	73.2 (40.8)	28.0 (11.3)	0.095

Numeric variables were expressed as mean with standard deviations (SD) and discrete outcomes as absolute and relative (%) frequencies. Other approaches were either open surgery alone or open and endoscopic surgery combined.

**Table 2:** Synthesis of included studies.

Author. Year	Sample Size (n)	Age (Years)	Follow up (Month)	Recurrence (n)	Recurrence Time (Month)	MINOR Score	MINOR Maximum Score
Lawson. 1995 [35]	7	57.3	54.0	3	NA	11	16
Bertrand. 2000 [22]	85	58.4	41.9	15	8	16	24
Lawson. 2003 [36]	13	NA	NA	3	NA	14	24
Dubin. 2005 [27]	6	NA	NA	5	13.3	14	24
Von Buchwald. 2005 [48]	13	61.0	37.0	0	9	16	24
Minovi. 2006 [39]	13	NA	NA	0	NA	14	24
Holzmann. 2007 [31]	9	NA	35.0	1	35	11	16
Woodworth. 2007 [53]	9	NA	40	4	NA	11	16
Sautter. 2007 [43]	5	55.0	NA	0	16.8	14	24
Kim. 2008 [34]	5	51.4	41.9	0	NA	16	24
Zhang. 2008 [51]	9	50.2	15.1	0	NA	15	24
Yoon. 2009 [49]	18	55.0	36.6	4	36	16	24
Dragonetti. 2011 [26]	8	NA	NA	1	NA	14	24
Lombardi. 2011 [37]	27	NA	NA	2	NA	14	24
Kamel. 2012 [32]	6	49.0	27.0	0	NA	16	24
Kim. 2012 [14]	22	54.1	41.0	6	32.6	16	24
Sciarretta. 2014 [44]	9	58.7	18.0	2	18	14	24
Promsopa. 2015 [42]	17	55.0	7.5	0	7.5	10	16
Adriaensen. 2015 [20]	20	53.7	47.6	2	42	16	24
Karligkiotis. 2015 [33]	5	48.0	40.6	0	NA	16	24
Akkari. 2016 [21]	12	NA	NA	3	23.6	9	16
Nygren. 2016 [40]	10	NA	NA	10	NA	9	16
Takahashi. 2016 [46]	10	58.4	39.5	0	NA	11	16
Adriaensen. 2016 [19]	17	NA	NA	3	26.6	14	24
Healy. 2016 [30]	26	56.9	96.0	4	32.4	16	24
Verillaud. 2016 [47]	27	58.0	40.0	2	40	16	24
Choi. 2019 [24]	23	NA	NA	6	37.76	9	16
Pietrobon. 2019 [41]	47	57.0	43.0	2	24	16	24
Coutinho. 2020 [25]	5	NA	NA	1	11.4	9	16
Ferrari. 2020 [28]	12	NA	NA	1	34	9	16
Lee. 2020 [12]	14	NA	NA	9	NA	14	24
Sham. 2020 [45]	29	55.0	56.4	12	39.8	16	24
Minni. 2021 [38]	21	NA	NA	3	NA	14	24
Glikson. 2022 [29]	17	NA	NA	3	NA	9	16
Yu. 2023 [50]	9	NA	NA	1	NA	14	24
Cho. 2024 [23]	13	60.6	NA	0	NA	9	16
Delaine. 2024 [52]	5	55.5	NA	3	23.8	9	16
SamimiArdestani. 2024 [54]	9	NA	NA	4	NA	9	16
Gaudioso. 2024 [16]	30	57	37	3	NA	16	24

For each study, first author, date of publication, sample size (number of patients per study), recurrence details, and MINORS score is given. NA; not available.

## Data Availability

Original data are available on request.

## Supplementary Materials

The following supporting information can be downloaded at: https://www.mdpi.com/article/10.3390/jpm15050183/s1, Supplementary Materials S1: MeSH equations.

## Abbreviations

The following abbreviations are used in this manuscript:

IP	Inverted Papilloma
MINORS	Methodological Index for Non-Randomized Studies
SWIM	Systematic Review without Meta-Analysis
EPOS	European Position Paper on Sinus

## References

[1] Wang M., Noel J.E. (2017). Etiology of Sinonasal Inverted Papilloma: A Narrative Review. World J. Otorhinolaryngol.-Head Neck Surg..

[2] Vorasubin N., Vira D., Suh J.D., Bhuta S., Wang M.B. (2013). Schneiderian Papillomas: Comparative Review of Exophytic, Oncocytic, and Inverted Types. Am. J. Rhinol. Allergy.

[3] Lisan Q., Laccourreye O., Bonfils P. (2016). Sinonasal Inverted Papilloma: From Diagnosis to Treatment. Eur. Ann. Otorhinolaryngol. Head Neck Dis..

[4] Elliot A., Marklund L., Håkansson N., Song H., Ye W., Stjärne P., Hammarstedt-Nordenvall L. (2017). Incidence of IP and Risk of Malignant Transformation in the Swedish Population 1960–2010. Eur. Arch. Oto-Rhino-Laryngol..

[5] Lawson W., Schlecht N.F., Brandwein-Gensler M. (2008). The Role of the Human Papillomavirus in the Pathogenesis of Schneiderian Inverted Papillomas: An Analytic Overview of the Evidence. Head Neck Pathol..

[6] Govindaraj S., Wang H. (2014). Does Human Papilloma Virus Play a Role in Sinonasal Inverted Papilloma?. Curr. Opin. Otolaryngol. Head Neck Surg..

[7] d’Errico A., Zajacova J., Cacciatore A., Baratti A., Zanelli R., Alfonzo S., Beatrice F. (2013). Occupational Risk Factors for Sinonasal Inverted Papilloma: A Case–Control Study. Occup. Environ. Med..

[8] Buchwald C., Franzmann M., Tos M. (1995). Sinonasal Papillomas: A Report of 82 Cases in Copenhagen County, Including a Longitudinal Epidemiological and Clinical Study. Laryngoscope.

[9] Kamel R.H., Khaled A., Abdelfattah A.F., Awad A.G. (2022). Surgical Treatment of Sinonasal Inverted Papilloma. Curr. Opin. Otolaryngol. Head Neck Surg..

[10] Russo C., Elefante A., Romano A., Cama A., Erra M., Ugga L., Brunetti L., Motta G., Califano L., Iengo M. (2021). A Multimodal Diagnostic Approach to Inverted Papilloma: Proposal of a Novel Diagnostic Flow-Chart. Curr. Probl. Diagn. Radiol..

[11] Kuan E.C., Wang E.W., Adappa N.D., Beswick D.M., London N.R., Su S.Y., Wang M.B., Abuzeid W.M., Alexiev B., Alt J.A. (2024). International Consensus Statement on Allergy and Rhinology: Sinonasal Tumors. Int. Forum Allergy Rhinol..

[12] Lee J.J., Roland L.T., Licata J.J., Orlowski H.L.P., Jiramongkolchai P., Piccirillo J.F., Kallogjeri D., Klatt-Cromwell C.N., Chernock R.D., Schneider J.S. (2020). Morphologic, Intraoperative, and Histologic Risk Factors for Sinonasal Inverted Papilloma Recurrence. Laryngoscope.

[13] Shohet J., Duncavage J. (1996). Management of the Frontal Sinus with Inverted Papilloma. Otolaryngol. Head Neck Surg..

[14] Kim D.-Y., Hong S.-L., Lee C.H., Jin H.-R., Kang J.M., Lee B.-J., Moon I.J., Chung S.-K., Rha K.-S., Cho S.H. (2012). Inverted Papilloma of the Nasal Cavity and Paranasal Sinuses: A Korean Multicenter Study. Laryngoscope.

[15] Krouse J.H. (2000). Development of a Staging System for Inverted Papilloma. Laryngoscope.

[16] Gaudioso P., Vinciguerra A., Verillaud B., Herman P. (2024). Management of Frontal Sinus and Frontal Recess Inverted Papilloma: Our Experience and Systematic Review. Acta Otorhinolaryngol. Ital..

[17] Campbell M., McKenzie J.E., Sowden A., Katikireddi S.V., Brennan S.E., Ellis S., Hartmann-Boyce J., Ryan R., Shepperd S., Thomas J. (2020). Synthesis without Meta-Analysis (SWiM) in Systematic Reviews: Reporting Guideline. BMJ.

[18] Slim K., Nini E., Forestier D., Kwiatkowski F., Panis Y., Chipponi J. (2003). Methodological Index for Non-Randomized Studies (Minors): Development and Validation of a New Instrument. ANZ J. Surg..

[19] Adriaensen G.F.J.P.M., Lim K.-H., Georgalas C., Reinartz S.M., Fokkens W.J. (2016). Challenges in the Management of Inverted Papilloma: A Review of 72 Revision Cases. Laryngoscope.

[20] Adriaensen G.F., van der Hout M.W., Reinartz S.M., Georgalas C., Fokkens W.J. (2015). Endoscopic Treatment of Inverted Papilloma Attached in the Frontal Sinus/Recess. Rhinology.

[21] Akkari M., Lassave J., Mura T., Gascou G., Pierre G., Cartier C., Garrel R., Crampette L. (2016). Atypical Presentations of Sinonasal Inverted Papilloma: Surgical Management and Influence on the Recurrence Rate. Am. J. Rhinol. Allergy.

[22] Bertrand B., Eloy P., Jorissen M., Rombaux P., Daele J., Boniver V., Collet S., Demanez J.P., Verheyden P.J., Bachert C. (2000). Surgery of Inverted Papillomas under Endoscopic Control. Acta Otorhinolaryngol. Belg..

[23] Cho S.W., Kim S.G., Han D.H., Kim H.J., Kim J.W., Kim D.Y., Rhee C.S., Won T.B. (2024). Treatment Outcome and Prognostic Factors of Inverted Papilloma Involving the Frontal Sinus. Laryngoscope Investig. Otolaryngol..

[24] Choi W.R., Lee B.J., Kim J.H. (2019). Long-Term Outcome Following Resection of Sinonasal Inverted Papillomas: A Single Surgeon’s Experience in 127 Patients. Clin. Otolaryngol..

[25] Coutinho G., Marques J., Leal M., Spratley J., Fernandes M.S., Santos M. (2020). Surgical Outcomes of Sinonasal Inverted Papilloma: A 17 Year Review. Braz. J. Otorhinolaryngol..

[26] Dragonetti A., Gera R., Sciuto A., Scotti A., Bigoni A., Barbaro E., Minni A. (2011). Sinonasal Inverted Papilloma: 84 Patients Treated by Endoscopy and Proposal for a New Classification. Rhinology.

[27] Dubin M.G., Sonnenburg R.E., Melroy C.T., Ebert C.S., Coffey C.S., Senior B.A. (2005). Staged Endoscopic and Combined Open/Endoscopic Approach in the Management of Inverted Papilloma of the Frontal Sinus. Am. J. Rhinol..

[28] Ferrari M., Schreiber A., Mattavelli D., Rampinelli V., Bertazzoni G., Tomasoni M., Gualtieri T., Nicolai P. (2020). How Aggressive Should Resection of Inverted Papilloma Be? Refinement of Surgical Planning Based on the 25-Year Experience of a Single Tertiary Center. Int. Forum. Allergy Rhinol..

[29] Glikson E., Dragonetti A., Soudry E., Rozendoren N., Alon E.E., Landsberg R., Schneider S., Bedrin L., Mozzanica F., Bulgheroni C. (2022). Is Intraoperative Margin Sampling Necessary in Inverted Papilloma Resection?. Eur. Arch. Otorhinolaryngol..

[30] Healy D.Y., Chhabra N., Metson R., Holbrook E.H., Gray S.T. (2016). Surgical Risk Factors for Recurrence of Inverted Papilloma. Laryngoscope.

[31] Holzmann D., Hegyi I., Rajan G.P., Harder-Ruckstuhl M. (2007). Management of Benign Inverted Sinonasal Papilloma Avoiding External Approaches. J. Laryngol. Otol..

[32] Kamel R.H., Abdel Fattah A.F., Awad A.G. (2012). Origin Oriented Management of Inverted Papilloma of the Frontal Sinus. Rhinology.

[33] Karligkiotis A., Pistochini A., Turri-Zanoni M., Terranova P., Volpi L., Battaglia P., Bignami M., Castelnuovo P. (2015). Endoscopic Endonasal Orbital Transposition to Expand the Frontal Sinus Approaches. Am. J. Rhinol. Allergy.

[34] Kim Y.M., Kim H.S., Park J.Y., Koo B.S., Park Y.H., Rha K.S. (2008). External vs. Endoscopic Approach for Inverted Papilloma of the Sino-Nasal Cavities: A Retrospective Study of 136 Cases. Acta Otolaryngol..

[35] Lawson W., Ho B.T., Shaari C.M., Biller H.F. (1995). Inverted Papilloma: A Report of 112 Cases. Laryngoscope.

[36] Lawson W., Kaufman M.R., Biller H.F. (2003). Treatment Outcomes in the Management of Inverted Papilloma: An Analysis of 160 Cases. Laryngoscope.

[37] Lombardi D., Tomenzoli D., Buttà L., Bizzoni A., Farina D., Sberze F., Karligkiotis A., Castelnuovo P., Nicolai P. (2011). Limitations and Complications of Endoscopic Surgery for Treatment for Sinonasal Inverted Papilloma: A Reassessment after 212 Cases. Head Neck.

[38] Minni A., Gera R., Bulgheroni C., Ralli M., Cialente F., Candelori F., Mevio N., Dragonetti A. (2021). Endoscopic Resection of Sinonasal Inverted Papilloma: A Multivariate Retrospective Analysis of Factors Affecting Recurrence and Persistence. Ear Nose Throat J..

[39] Minovi A., Kollert M., Draf W., Bockmühl U. (2006). Inverted Papilloma: Feasibility of Endonasal Surgery and Long-Term Results of 87 Cases. Rhinology.

[40] Nygren A., Kiss K., von Buchwald C., Bilde A. (2016). Rate of Recurrence and Malignant Transformation in 88 Cases with Inverted Papilloma between 1998-2008. Acta Otolaryngol..

[41] Pietrobon G., Karligkiotis A., Turri-Zanoni M., Fazio E., Battaglia P., Bignami M., Castelnuovo P. (2019). Surgical Management of Inverted Papilloma Involving the Frontal Sinus: A Practical Algorithm for Treatment Planning. Acta Otorhinolaryngol. Ital..

[42] Promsopa C., Thanahirunrojh S. (2015). Surgical Outcomes of Sinonasal Inverted Papillomas in Songklanagarind Hospital. J. Med. Assoc. Thai.

[43] Sautter N.B., Citardi M.J., Batra P.S. (2007). Minimally Invasive Resection of Frontal Recess/Sinus Inverted Papilloma. Am. J. Otolaryngol..

[44] Sciarretta V., Fernandez I.J., Farneti P., Pasquini E. (2014). Endoscopic and Combined External-Transnasal Endoscopic Approach for the Treatment of Inverted Papilloma: Analysis of 110 Cases. Eur. Arch. Otorhinolaryngol..

[45] Sham C.L., van Hasselt C.A., Chow S.M.W., Lee D.L.Y., Cho R.H.W., Woo J.K.S., Tong M.C.F. (2020). Frontal Inverted Papillomas: A 25-Year Study. Laryngoscope.

[46] Takahashi Y., Shoji F., Katori Y., Hidaka H., Noguchi N., Abe Y., Kakuta R.K., Suzuki T., Suzuki Y., Ohta N. (2016). Endoscopic Surgical Management of Sinonasal Inverted Papilloma Extending to Frontal Sinuses. Otolaryngol. Pol..

[47] Verillaud B., Le Clerc N., Blancal J.P., Guichard J.P., Kania R., Classe M., Herman P. (2016). Mucocele Formation after Surgical Treatment of Inverted Papilloma of the Frontal Sinus Drainage Pathway. Am. J. Rhinol. Allergy.

[48] Von Buchwald C., Larsen A.S. (2005). Endoscopic Surgery of Inverted Papillomas under Image Guidance—A Prospective Study of 42 Consecutive Cases at a Danish University Clinic. Otolaryngol. Head Neck Surg..

[49] Yoon B.N., Batra P.S., Citardi M.J., Roh H.J. (2009). Frontal Sinus Inverted Papilloma: Surgical Strategy Based on the Site of Attachment. Am. J. Rhinol. Allergy.

[50] Yu S., Grose E., Lee D.J., Wu V., Pellarin M., Lee J.M. (2023). Evaluation of Inverted Papilloma Recurrence Rates and Factors Associated Recurrence after Endoscopic Surgical Resection: A Retrospective Review. J. Otolaryngol. Head Neck Surg..

[51] Zhang L., Han D., Wang C., Ge W., Zhou B. (2008). Endoscopic Management of the Inverted Papilloma with Attachment to the Frontal Sinus Drainage Pathway. Acta Otolaryngol..

[52] Delaine E., Gorostidi F., Guilcher P., Lambercy K., Litzistorf Y., Bron L., Reinhard A. (2024). Risk Factors for Recurrence after Surgical Resection of Sinonasal Inverted Papilloma. Int. Arch. Otorhinolaryngol..

[53] Woodworth B.A., Bhargave G.A., Palmer J.N., Chiu A.G., Cohen N.A., Lanza D.C., Bolger W.E., Kennedy D.W. (2007). Clinical Outcomes of Endoscopic and Endoscopic-Assisted Resection of Inverted Papillomas: A 15-Year Experience. Am. J. Rhinol..

[54] SamimiArdestani S. (2024). The Characteristics of Sinonasal Inverted Papilloma and Recurrence Factors: An Analysis of 207 Cases. World J. Otorhinolaryngol. Head Neck Surg..

[55] Busquets J.M., Hwang P.H. (2006). Endoscopic Resection of Sinonasal Inverted Papilloma: A Meta-Analysis. Otolaryngol.-Head Neck Surg..

[56] Kim J.S., Kwon S.H. (2017). Recurrence of Sinonasal Inverted Papilloma Following Surgical Approach: A Meta-Analysis. Laryngoscope.

[57] Bastier P.L., de Gabory L. (2016). Design and Assessment of an Anatomical Diagram for Sinonasal Malignant Tumour Resection. Rhinology.

[58] Wang Y., An Y., Zhao C., Dong R., Cheng F. (2020). Attachment-Oriented Endoscopic Treatment of Inverted Papilloma Involving the Frontal Sinus/Recess. J. Craniofac. Surg..

[59] Yan Y., Liu Y., Tao J., Li Z., Qu X., Guo J., Xian J. (2022). Preoperative Prediction of Malignant Transformation of Sinonasal Inverted Papilloma Using MR Radiomics. Front. Oncol..

[60] Lawson W., Ho Y. (2016). Open Frontal Sinus Surgery: A Lost Art. Otolaryngol. Clin. N. Am..

