# Supplementary material for: Endoscopic Sinus Surgery in Frontal Sinus Inverted Papilloma: A Systematic Review
Source: J Pers Med. 2025 May 2;15(5):183. doi: 10.3390/jpm15050183 (PMC12113324; doi:10.3390/jpm15050183)
Supplement: Supplementary file 1 [file jpm-15-00183-s001.zip › jpm-3560517-supplementary.pdf]

Pubmed	1167 results<br><br>(("Papilloma, Inverted"[Mesh] OR (Papilloma*[Tiab] AND (Invert*[Tiab] OR Schneiderian[Tiab]))) AND ("Frontal Sinus"[Mesh] OR "Paranasal Sinus Neoplasms"[Mesh] OR "Paranasal Sinuses"[Mesh] OR "Neoplasm Recurrence, Local"[Mesh] OR (Sinus*[Tiab] AND Frontal[Tiab]) OR (Agger[Tiab] AND Nasi[Tiab] AND Cell*[Tiab]) OR (Frontal[Tiab] AND Recess*[Tiab]) OR ((Neoplasm*[Tiab] OR cancer*[Tiab]) AND Paranasal[Tiab] AND Sinus*[Tiab]) OR ((Nasal[Tiab] AND Sinus*[Tiab]) OR (Ostiomeatal[Tiab] AND (Compl*[Tiab] OR Unit*[Tiab])) OR (Sinonasal[Tiab] AND Tract*[Tiab]) OR (Supraorbital[Tiab] AND Ethmoid[Tiab] AND Cell*[Tiab])) OR (Neoplasm*[Tiab] AND Recurrence*[Tiab] AND (Local[Tiab] OR Locoregional[Tiab])) OR "Paranasal sinus*"[Tiab]) AND (English[lang] OR French[lang] OR Spanish[lang] OR Italian[lang] OR German[lang] OR Portuguese[lang]))
WoS	852 results<br><br>TS=((Papilloma* AND (Invert* OR Schneiderian))) AND TS=(((Sinus* AND Frontal) OR (Agger AND Nasi AND Cell*) OR (Frontal AND Recess*) OR ((Neoplasm* OR cancer*) AND Paranasal AND Sinus*) OR ((Nasal AND Sinus*) OR (Ostiomeatal AND (Compl* OR Unit*)) OR (Sinonasal AND Tract*) OR (Supraorbital AND Ethmoid AND Cell*)) OR (Neoplasm* AND Recurrence* AND (Local OR Locoregional)) OR "Paranasal sinus*")) AND LA=(English OR French OR Spanish OR Italian OR German OR Portuguese)
Embase	892 results<br><br>papilloma*:ti,ab,kw AND (invert*:ti,ab,kw OR schneiderian:ti,ab,kw) AND (sinus*:ti,ab,kw AND frontal:ti,ab,kw OR (agger:ti,ab,kw AND nasi:ti,ab,kw AND cell*:ti,ab,kw) OR (frontal:ti,ab,kw AND recess*:ti,ab,kw) OR ((neoplasm*:ti,ab,kw OR cancer*:ti,ab,kw) AND paranasal:ti,ab,kw AND sinus*:ti,ab,kw) OR (nasal:ti,ab,kw AND sinus*:ti,ab,kw) OR (ostiomeatal:ti,ab,kw AND (compl*:ti,ab,kw OR unit*:ti,ab,kw)) OR (sinonasal:ti,ab,kw AND tract*:ti,ab,kw) OR (supraorbital:ti,ab,kw AND ethmoid:ti,ab,kw AND cell*:ti,ab,kw) OR (neoplasm*:ti,ab,kw AND recurrence*:ti,ab,kw AND (local:ti,ab,kw OR locoregional:ti,ab,kw)) OR 'paranasal sinus*':ti,ab,kw) AND (english:la OR french:la OR spanish:la OR italian:la OR german:la OR portuguese:la)
CENTRAL and SciELO	8 + 6 results<br><br>Trials matching (Papilloma* AND (Invert* OR Schneiderian)) in Title Abstract Keyword AND ((Sinus* AND Frontal) OR (Agger AND Nasi AND Cell*) OR (Frontal AND Recess*) OR ((Neoplasm* OR cancer*) AND Paranasal AND Sinus*) OR ((Nasal AND Sinus*) OR (Ostiomeatal AND (Compl* OR Unit*)) OR (Sinonasal AND Tract*) OR (Supraorbital AND Ethmoid AND Cell*)) OR (Neoplasm* AND Recurrence* AND (Local OR Locoregional)) OR (Paranasal NEAR sinus*)) in Title Abstract Keyword - (Word variations have been searched)
